# Book Notices

**Published:** 1880-03-20

**Authors:** 


### *BOOK NOTICES.*

A SYSTEM OF MEDICINE. Edited by J. Russell Reynolds, M.D., F.R.S., Fellow of the Royal College of Physicians of London; Fellow of the Imperial Leopold-Carolina Academy of London; Fellow of University College, London; Professor of the Principles and Practice of Medicine in University College; Physician to University College Hospital; Examiner in Medicine to the University of London. With Numerous Additions and Illustrations, by Henry Hartshorne, A M., M.D., Fellow of the College of Physicians of Philadelphia; formerly Professor of Practice of Medicine in Medical Department of Pennsylvania College, and Physician to the Episcopal Hospital of Philadelphia; lately Professor of Hygiene in the University of Pennsylvania, and Professor of Hygiene and Diseases of Children in the Woman's Medical College of Pennsylvania, etc. In Three Volumes. Philadelphia: Henry C. Lea. 1880.

We have before us the first and second volumes of this great work—aggregating nearly two thousand large double-column octavo pages, neatly printed, with full and separate index to each volume, and containing numerous illustrations.

The work has been gotten up not by a single individual, but by leading, eminent minds, selected for their special ability and acquaintance with the particular subjects treated, and presents, therefore, an able review of modern British medicine in all departments. Such a work must prove highly valuable and instructive, and for comparison with American medical works of the same kind, cannot fail to interest exceedingly the professional reader and student in this country.

We have examined the volumes before us with much interest, and recommend them to our readers as books of very great merit. The third and last volume, we learn, will be out in the course of the present month, and the work complete may be obtained at the house of Henry C. Lea, Philadelphia.

REPORT OF THE REVISION OF THE U. S. PHARMACOPOEIA, Preliminary to the Convention of 1880. Being a Rough Draft of the General Principles, Titles, and Working Formulæ proposed for the next Pharmacopoeia. Prepared and Compiled by Charles Rice, Chairman of the Committee of the American Pharmaceutical Association. New York. 1880.

This preliminary report of the Revision Committee must prove highly interesting to the profession, which has so long anxiously desired a revision of the United States Pharmacopoeia.

The improvements suggested are many and valuable. They relate to *Language*, *Alphabetical Arrangement*, *Synonyms*, *Cross References*, *Descriptions of Drugs and Chemicals*, *Formulæ*, *Expressions of Quantity in parts by Weight*, etc. Certain articles are to be wholly dropped as obsolete and useless. Upon this point, we hope the dropping will not be too sweeping. The student of Materia Medica often desires to know what a thing is, as a matter of information merely. Many articles also have been revived and brought again into use after lying for a long while neglected, and many articles ignored by one class of practitioners are efficiently used by another class.

The *new tables* proposed are all useful. The table of *Poisons and their Antidotes* should not be dropped, as some have suggested.

We are pleased with the *New Additions* of remedies. We trust that all the agents brought into use of late years will be incorporated. The properties of many, it is true, are not definitely settled yet; it is well that the properties ascribed to them be briefly mentioned, that they may be tested by the profession. There are also in domestic practice certain familiar plants which are not in our present Dispensatory. Of these, we mention the common *heart leaf*, the botanical name of which is not given in King's Eclectic Dispensatory, or any other work on Materia Medica that we know of.

The substitution of *vaseline* for lard as a vehicle for ointments we trust will be considered. It never becomes rancid. Its consistence may be increased by the addition of white wax, as follows:

Vaseline.................................. 16 ounces.
White wax.................................. 8 ounces.
Melt with gentle heat.

This cerate will not become rancid, is neither too hard for cold or too soft for warm weather, and may be substituted for lard in all cases where that agent is used.

